# Why Some Patients Choose Nutritional Therapy over Medications and Surgery in Obesity Care

**DOI:** 10.3390/nu18060950

**Published:** 2026-03-17

**Authors:** Hilary C. Craig, Dalal Alaseed, Ebaa Al Ozairi, Werd Al-Najim, Carel W. le Roux

**Affiliations:** 1 Metabolic Medicine, St. Vincent’s University Hospital, D04 T6F4 Dublin, Ireland; 2 Clinical Research and Clinical Trial Unit, Dasman Diabetes Institute, Dasman P.O. Box 1180, Kuwait; 3 Diabetes Complications Research Centre, UCD Conway Institute of Biomedical and Biomolecular Research, School of Medicine, University College Dublin, Belfield, D04 V1W8 Dublin, Ireland

**Keywords:** obesity, nutritional therapies, people living with obesity, treatment options, patient preferences

## Abstract

Introduction: Obesity is a well-established risk factor for numerous chronic diseases, including type 2 diabetes, cardiovascular disease, and certain cancers. Obesity-related complications can be managed through nutritional therapy, pharmacotherapy, and surgical interventions, each capable of achieving weight loss of over 10%. Understanding patient preferences and the factors that influence treatment choices is crucial to enhancing adherence and effectiveness. This sub-study aimed to identify the factors shaping patient preferences for nutritional therapies in the context of available pharmacological and surgical options. Methods: A participatory action study recruited 43 patients aged 18–75 years with a BMI greater than 35 kg/m^2^ and obesity-related complications, including metabolic dysfunction, diabetes, hypertension, and chronic kidney disease. Participants viewed a 60-min informational video outlining treatment options before taking part in one-to-one interviews. Data were analysed using reflective thematic analysis. Results: This sub-study focuses on patients who expressed distinct attitudes toward nutritional therapy. Of the participants, 47% preferred nutritional therapy, 41% chose pharmacotherapy alone, and 6% selected a combination of pharmacotherapy and nutritional therapy. Five themes emerged to explain the preference for nutritional therapy: patient satisfaction, the personalised approach, effectiveness, empowerment, and side effects. Discussion: Nutritional therapies were still the most popular choice of many patients, suggesting there remain unmet needs of patients and that it should not be assumed that large majorities of patients with obesity only want pharmacotherapies or surgical therapies. Conclusion: Ensuring patients receive comprehensive information and regular guidance from nutritional experts is likely to further strengthen engagement.

## 1. Introduction

Obesity is a complex, multifactorial disease that contributes to chronic complications such as diabetes, hypertension, chronic kidney disease, liver disease, and many other obesity complications. This situation adversely affects patients’ quality of life, reduces life expectancy, and imposes a significant financial burden on healthcare delivery. The anticipated rise in obesity-related diseases will impose an economic strain on healthcare resources [1,2,3]. Evidence-based treatment options, including nutritional therapies, pharmacotherapies, and surgical therapies that achieve a weight loss of 10% or more, can lead to significant health improvements for those with obesity-related complications [4].

Despite the availability of effective treatments, international research consistently shows that many individuals with obesity do not progress to pharmacological or surgical options, even when clinically indicated. Studies have identified multiple factors shaping treatment preferences, including perceptions of risk and invasiveness, previous negative experiences with healthcare, limited health literacy, and concerns about long-term safety and side effects [5,6,7]. Behavioural and nutritional approaches are often preferred because they are perceived as safer, more familiar, and more aligned with personal values, even when their effectiveness may be limited without sustained professional support. System-level barriers, such as long waiting times, fragmented services, and high out-of-pocket costs, further restrict access to specialist obesity care and influence how patients evaluate available treatment pathways.

Existing research has highlighted the importance of patient-centred care, trust in healthcare professionals, and continuity of support in shaping engagement with obesity treatment [8,9]. However, relatively few studies have explored why patients with obesity-related complications actively choose nutritional therapy over pharmacotherapy or surgery when all options are presented concurrently. Understanding these preferences is essential for designing responsive, equitable, and person-centred obesity services.

Previously, it was demonstrated that when patients with a BMI greater than 35 kg/m^2^ and obesity-related complications are given the option to choose between nutritional therapy, pharmacotherapy, or surgical procedures, several factors influence their treatment decisions. These factors include: (1) systemic issues related to healthcare, such as accessibility and cost; (2) a lack of knowledge and feeling unheard; (3) experiences with formal services that reflect inadequate support and information; and (4) the emotional and physical consequences of obesity, particularly the fear of potential medical complications [10].

This sub-study formed part of a larger participatory action research project that explored patient experiences, treatment preferences, and decision-making in obesity care through a combination of semi-structured interviews, photovoice, and World Café sessions. The overarching study design and methodology have been published previously [11,12]. The parent study employed an iterative, collaborative design to capture patient perspectives across multiple qualitative modalities and to inform the development of more person-centred approaches to obesity management. The present analysis focuses specifically on understanding why 47% of participants with obesity-related complications selected nutritional therapy as their preferred intentional weight-loss strategy. By capturing the voices, values, and priorities of individuals living with this chronic and debilitating disease, the study aims to inform the development of more person-centred clinical services and support more effective shared decision-making in obesity care.

## 2. Methods

To gain deeper insight into patient perspectives, we employed participatory action research (PAR), an approach that emphasises collaboration with participants by sharing findings and working together to promote positive change [13]. We aimed to strengthen understanding of obesity and its treatment options, ensuring that patient voices were captured and used to advocate for improvement.

Data collection was conducted over two years and analysed using reflective thematic analysis. Three complementary methodologies were employed: one-on-one interviews, which provided in-depth accounts of patients’ experiences and preferences; photovoice, which encouraged participants to capture images reflecting their personal perspectives; and World Café sessions, which facilitated group discussions in a conversational format, allowing participants to share their ideas collectively. This sub-study draws specifically on the semi-structured interviews and the photovoice component of the wider project. Only participants who completed these two components contributed data to the present analysis. Although World Café sessions formed part of the overall PAR process, they were not included as primary data sources for this manuscript. Instead, outputs from the World Café were used solely as contextual reference points during triangulation, helping situate the interview and photovoice findings within the broader project. They did not contribute raw data to coding or theme development.

The interviews provided in-depth accounts of patient experiences and treatment preferences, while photovoice enabled participants to capture images reflecting their personal perspectives. World Café sessions facilitated group discussions in a conversational format, but as noted, were not analysed directly for this manuscript.

All interviews were conducted by an experienced obesity healthcare professional. The interviewer was a senior healthcare professional working in oncology services, with a PhD in Public Health and additional training in health care ethics, law, and management. Their research expertise includes obesity and patient-centred care, and they held no clinical role in the care of participating patients, thereby ensuring neutrality regarding treatment preferences, and the additional healthcare professionals involved in the broader study contributed to their capacity as qualitative research experts rather than as treating clinicians.

Participants were not previously known to the researcher, and no therapeutic relationship existed, which helped to minimise potential bias. Reflexive notes were maintained throughout to monitor potential influence on questioning and interpretation. A semi-structured interview guide was developed collaboratively by the research team, [14], drawing on prior studies and insights from the Stratification of Obesity Phenotypes to Optimise Future Obesity Therapy (SOPHIA) project. Before the interviews, the participants viewed a 60-min informational video that presented all intentional weight-loss treatment options. The educational video was developed and delivered by researchers at University College Dublin with expertise in obesity care and patient-centred communication. Each treatment option was then presented by a clinical expert in that area, including a physician specialising in metabolic medicine, a bariatric surgeon, and a specialist dietitian.

Lifestyle changes remain foundational to all obesity treatments, including nutritional therapies, pharmacotherapies, and surgical therapies. Lifestyle changes as defined in this study include healthy eating (reductions in fat and refined carbohydrates) and healthy exercise (resistance and aerobic exercise). In contrast, nutritional therapies included specific treatments for obesity aimed to reduce appetitive behaviour such as (but not exclusive to) the zone diet, keto diet, intermittent fasting, etc. This description reflects the established clinical guidelines, which position dietary change and physical activity as foundation components of obesity care.

Interviews began with broad questions before progressing to more specific inquiries about treatment preferences, allowing exploration of motivations and decision-making factors. Each session lasted between 30 and 45 min. This sub-study focuses on data relating to preferences for nutritional therapies, derived from the interviews and photovoice methods.

Interview data were analysed using reflexive thematic analysis following Braun and Clarke’s six-phase approach [14]. Transcripts were read repeatedly for familiarisation, and inductive coding was undertaken to privilege participants’ own language and meanings. Codes were then organised into preliminary themes, which were reviewed and refined through iterative team discussions. A second experienced qualitative researcher independently coded a subset of transcripts, and differences were resolved through consensus. Themes were evaluated for coherence, distinctiveness, and their ability to represent meaningful patterns across the dataset. Triangulation across interviews, photovoice, and World Café data further enhanced credibility and reduced the potential influence of individual researcher interpretation.

Participants were not enrolled in or exposed to any active treatment (e.g., nutritional therapy, pharmacotherapy, or surgery) as part of this study. The interviews focused on participants’ perceptions of the effectiveness of different weight-management options, informed by their prior personal experiences and by the standardised educational video viewed before the interview. Participants were asked to discuss their previous experiences with weight-management approaches and their views on the perceived effectiveness of the treatment options presented. The study did not involve delivering or evaluating any treatment; rather, it explored participants’ beliefs, expectations, and decision-making processes.

## 3. Recruitment

Purposeful sampling was used to recruit 43 patients with obesity-related complications. As Creswell (2012) notes, purposeful sampling involves the intentional selection of individuals and sites that are “information-rich,” thereby enabling a deeper understanding of the phenomenon under study [13]. In qualitative research, adequacy of sample size is guided by the principle of reaching saturation in emerging themes. Recruitment occurred across two clinical sites in Dublin, Ireland, and multiple specialist clinics, including metabolic dysfunction-associated steatotic liver disease, diabetes, hypertension, and chronic kidney disease services. Although some of these clinics were located within hospitals without dedicated obesity services, they provided access to a diverse cohort of patients living with obesity in different clinical contexts. No new concepts emerged across interviews, photovoice submissions, and World Café discussions. The convergence of themes across these three methodologies further confirmed that additional recruitment was unlikely to yield substantively new insights. Participants were eligible if they were adults aged 18–75 years, had a body mass index greater than 35 kg/m^2^, and had an obesity-related complication such as diabetes, liver disease, or kidney disease. Participant characteristics are presented in Table 1. Exclusion criteria included individuals younger than 18 or older than 75 years, those with dementia or Alzheimer’s disease, and those without verbal communication skills. A notable strength of this study lies in its participatory action design, which integrates multiple data sources, enhances the depth and credibility of findings and supports validation of findings through triangulation in future projects.

## 4. Data Analysis

The first author developed a coding framework informed by previous research and the interview transcripts [15]. We used a hybrid deductive–inductive analytical approach. Sensitising concepts from the study aims and existing literature informed the initial coding framework, while inductive coding allowed new insights to emerge directly from participants’ accounts. This combination ensured that the final themes reflected both prior knowledge and data-driven interpretations. All interviews and discussions were recorded and transcribed verbatim, with transcripts anonymised before being entered into MAXQDA 2022 Plus software to support systematic coding. Reflective thematic analysis was then undertaken by the first author, using an inductive approach to identify and refine themes and sub-themes emerging from the data. Interpretation was guided by a socio-cultural lens, allowing exploration of the factors shaping participants’ choices, including their motivations and the influence of obesity-related complications. In addition, content analysis was performed to quantify the proportion of participants who expressed intentions to pursue specific obesity treatments. Codes and themes were further refined through iterative discussions among all authors, a process that encouraged reflexivity and dialogue and ultimately led to consensus. Ethical approval for the study was granted by the Human Research Ethics Committee—Sciences (HREC) at University College Dublin, Ireland, on 6 August 2021.

## 5. Results

The first theme to emerge was patient satisfaction. Most patients reported significantly higher levels of satisfaction with nutritional therapy compared to other obesity treatments. They appreciated the holistic approach of nutritional therapy, which not only alleviates symptoms but also enhances overall well-being. Patients felt that this comprehensive method addressed their physical health needs, leading to a more fulfilling treatment experience.

“*Well, my preference would be nutrition, I think overall. As I told you the benefit, I can see there’s several fold and overall lifestyle change and more focused, focused idea of how to live properly and how to look after yourself slightly better, I think that I would benefit from it on many fronts*.”

Patient with MASLD

The second theme was effectiveness. Patients who prefer nutritional therapies observed improvements in their symptoms and overall health after undergoing nutritional therapy. These patients felt nutritional therapies were particularly effective in managing complications of obesity. The reduction in symptoms and the improved quality of life experienced by these patients underscored the perception of efficacy of nutritional therapy. Additionally, many of these patients reported a decreased reliance on medication, further highlighting the desirability of nutritional therapies.

The third theme was side effects. Another compelling reason for patients’ preference for nutritional therapy was the minimal associated side effects. Unlike other treatments, which often come with various adverse reactions, nutritional therapy was generally well-tolerated. The absence of significant side effects makes nutritional therapies a safer option for long-term management of obesity, allowing these patients to adhere to their treatment regimen without fear of harmful consequences.

“*Surgery and medication, I would not know enough about. I suppose I’d be in a lack of knowledge about it. that I wouldn’t know enough, and maybe, it’s a big jump. It’s a big leap into the unknown. You know if it didn’t work or if there were side effects, you know. Are you going to be on the wrong side of sense with it? I just don’t know enough about it. I’m very respectful of medication, but I only take what I have to take. That sounds terrible, but from a cardiac point of view, I’m putting pressure on that. I’d be very doubtful of it. Everything has side effects. And obviously I’d like more knowledge and maybe more research into all of these things.*”

Patient with MASLD

The fourth theme was the personalised approach associated with nutritional therapies as it provides a highly personalised treatment plan tailored to the individual needs of each patient. This customisation is highly valued, as it addresses unique dietary preferences. The personalised approach ensures that patients receive the most appropriate and effective treatment for their specific conditions, leading to better outcomes and increased satisfaction.

“*My expectation for nutrition would be a different system where everyone has access to it. It’s very difficult to get a nutritionist, never mind in the community; hospitals don’t get it! Dietician, I mean, if hospitals cannot get a dietitian, how is John and Jane or whatever here and say in Portlaoise going to get it? Not a chance. But I will tell you what would work. When you’re obese, and you really want to change, but you don’t have any effect because you are too weak mentally or otherwise, you need two things: One, you need help from outside people; you do need help from outside people. So, it will be great if communities had a centre where there is a dedicated person, or two, to go to. Two, help people with obesity and it would be, it will be an ongoing process because the chronic problem has to be an ongoing process.*”

Patient with MASLD

The final theme was empowerment. These patients felt more in control of their health through nutritional therapy. The emphasis on education and self-management empowered them to make informed decisions about their treatment. By understanding the impact of nutrition on their health, these patients can take proactive steps toward improving their conditions. This sense of empowerment and autonomy significantly contributes to their preference for nutritional therapy.

“*Learning about nutrition is the most important one, and it’s something that it’s a skill you have for life. If you get surgery, even though you still have the same effects, you’re not learning what the correct foods to put into your body and why*.”

Patient with CKD

**Figure 1:**
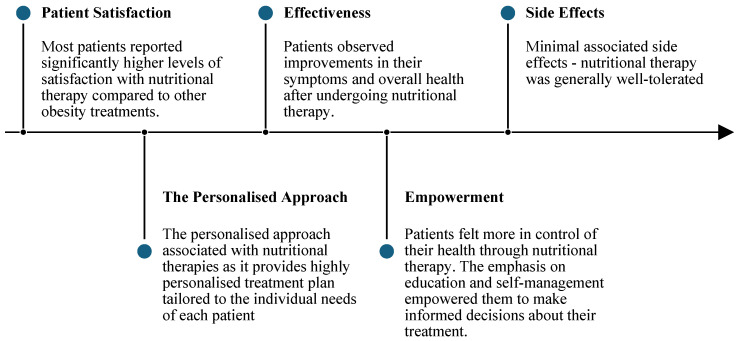
Themes.

The full list of themes is outlined in Figure 1.

Some participants noted long waiting times and the cost of private care as contextual challenges within the healthcare system, though these factors did not appear to shape their treatment choices.

## 6. Discussion

Obesity treatment is complex and requires sustained professional support, with nutritional interventions remaining central to management [16]. Adeola et al. (2023) highlighted significant individual variation in weight-loss responses to dietary strategies and argued that outcomes could be improved through tailored nutrition plans, proposing personalised nutrition profiles to better match individual needs [17].

Participants in our study reported substantial barriers to accessing obesity-trained healthcare professionals. Long waiting times, often extending over years, left patients with little guidance, while the cost of private care was prohibitive. Structural barriers to accessing obesity care are well documented, with long waiting lists, fragmented services, and high out-of-pocket costs consistently identified as limiting timely engagement with evidence-based treatment [18]. Our findings align with this work, illustrating how system-level constraints shape not only access but also patients’ sense of agency and treatment expectations. Despite these challenges, regular contact with health experts was regarded as essential for building trust, sustaining motivation, and receiving tailored advice. Patients valued holistic care from doctors or nurses and emphasised the importance of ongoing collaboration in refining dietary plans alongside psychological and medical support, consistent with evidence emphasising the importance of multidisciplinary, relationship-based care in obesity management [19].

Many expressed hope that, with sufficient guidance, nutritional therapies could help them manage their condition independently. This aligns with Nothwehr et al. (2013) and Snyder et al. (1991), who conceptualise hope as comprising “agency”—belief in one’s ability to achieve goals—and “pathways”—the perception of strategies to overcome obstacles [20,21]. Prior studies have shown that patients frequently favour behavioural or nutritional strategies because they are perceived as safer, more controllable, and more compatible with personal values compared with pharmacotherapy or surgery [22,23,24]. This resonates with our participants’ emphasis on low-risk, patient-centred approaches and their desire for interventions that support long-term self-management.

Concerns about pharmacological and surgical options further shaped preferences. To increase the penetrance of these modalities if needed, healthcare providers will need to understand the previous negative experiences with medications, fear of side effects, and reluctance to begin new regimens, as these contributed to the appeal of nutritional approaches. Surgery was often perceived as a drastic and risky measure, reinforcing anxieties about complications. For some, difficulty interpreting conflicting information influenced how they evaluated different treatment pathways. These factors may have contributed to the relative appeal of nutritional strategies, shaping preferences in ways that extend beyond perceptions of clinical effectiveness. Improving health literacy was seen as critical, particularly in discerning information relevant to individual circumstances. The central role of health literacy in shaping obesity treatment decisions is well established, with limited understanding of treatment options associated with lower uptake of pharmacological and surgical interventions [25,26]. Participants’ difficulty navigating conflicting information reflects these broader challenges and underscores the need for clear, personalised communication.

This sub-study sought to understand why nutritional therapies were preferred over pharmacotherapies and surgery as intentional weight-loss strategies. We considered potential subthemes during analysis; however, as they did not offer additional conceptual insight beyond the overarching themes, they were not reported separately.

Effectiveness in obesity management should be measured not only by weight reduction but by broader health gains, recognising that weight loss often plateaus and relapse of obesity is common if treatment is stopped. While some participants questioned the efficacy of nutritional approaches, they favoured them as a low-risk means of improving disease management. Adherence and success are greatest when nutritional interventions are personalised and supported by counselling, structured support systems, and physical activity recommendations. These findings indicate that participants valued patient-centred care and described tailored dietary changes, supported over time, as important in helping them navigate the multifaceted nature of obesity.

## 7. Strengths and Limitations

A key strength of this study was its use of participatory action research (PAR) [13], which positioned participants as equal partners and valued their lived experiences rather than treating them as passive subjects. Unlike traditional approaches that often overlook those most affected, PAR begins with participants’ realities and fosters inclusive, democratic engagement. Its collaborative nature proved particularly effective in exploring complex health experiences, enabling deeper insight through qualitative interviews and triangulated data. By balancing methodological rigour with participant empowerment, PAR generated valid findings while contributing to more responsive and impactful practices. The primary limitation of this study reflects the inherent constraints of qualitative research, in that the findings apply only to the participants involved. In addition, although the informational video was designed to provide neutral, factual context about obesity treatments, its use prior to the interviews may have shaped how participants framed their views; therefore, we acknowledge this as a potential influence on participants’ responses. Nevertheless, the themes that emerged can inform future qualitative and quantitative studies, thereby enhancing understanding of the research question.

## 8. Conclusions

This sub-study indicates that nutritional therapy is viewed by participants as a potentially valuable option within obesity management, both as an alternative and as a complement to other treatments. Its holistic nature, low risk of adverse effects, and capacity to empower patients through personalised care resonate strongly with those seeking sustainable and dignified solutions. By fostering autonomy and aligning with patients’ values and lived experiences, nutritional therapies address not only physical health but also psychological well-being. These findings underscore the importance of embedding patient-centred strategies within obesity care and demonstrate the potential of nutritional therapy to improve outcomes in ways that are both effective and meaningful for those with obesity.

## Figures and Tables

**Table 1:** Characteristics of Participants.

Age	Sex
18–75 years	Eighteen Males
	Twenty-Five Females
**Health Complications**	
40% of participants had Metabolic Dysfunction-Associated Steatotic Liver Disease (MASLD)	
28% of participants had Type 2 Diabetes (T2DM)	
14% of participants had hypertension	
26% of participants had Chronic Kidney Disease (CKD)	

## Data Availability

The datasets generated during and/or analysed during the current study are available from the corresponding author upon reasonable request.
